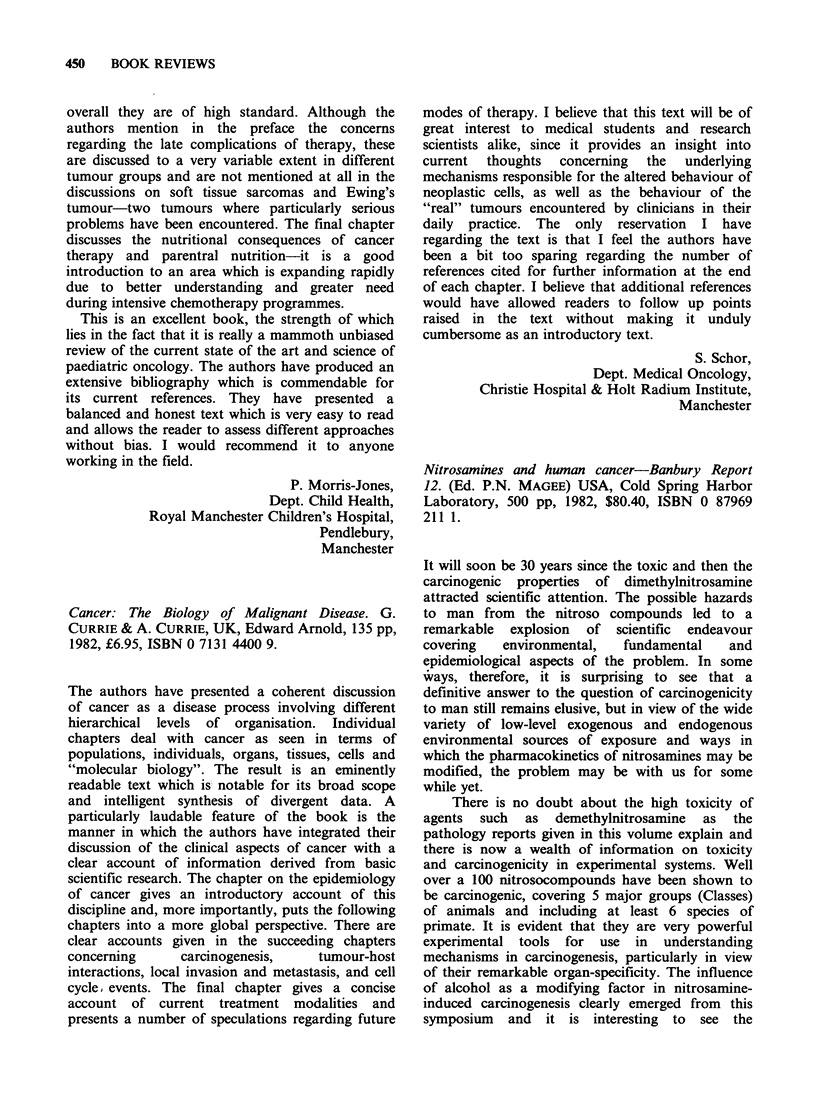# Cancer: The Biology of Malignant Disease

**Published:** 1983-09

**Authors:** S. Schor


*Cancer: The Biology of Malignant Disease.* G. CURRIE & A. CURRIE, UK, Edward Arnold, 135 pp, 1982, £6.95, ISBN 0 7131 4400 9.

The authors have presented a coherent discussion of cancer as a disease process involving different hierarchical levels of organisation. Individual chapters deal with cancer as seen in terms of populations, individuals, organs, tissues, cells and "molecular biology". The result is an eminently readable text which is notable for its broad scope and intelligent synthesis of divergent data. A particularly laudable feature of the book is the manner in which the authors have integrated their discussion of the clinical aspects of cancer with a clear account of information derived from basic scientific research. The chapter on the epidemiology of cancer gives an introductory account of this discipline and, more importantly, puts the following chapters into a more global perspective. There are clear accounts given in the succeeding chapters concerning carcinogenesis, tumour-host interactions, local invasion and metastasis, and cell cycle events. The final chapter gives a concise account of current treatment modalities and presents a number of speculations regarding future modes of therapy. I believe that this text will be of great interest to medical students and research scientists alike, since it provides an insight into current thoughts concerning the underlying mechanisms responsible for the altered behaviour of neoplastic cells, as well as the behaviour of the "real" tumours encountered by clinicians in their daily practice. The only reservation I have regarding the text is that I feel the authors have been a bit too sparing regarding the number of references cited for further information at the end of each chapter. I believe that additional references would have allowed readers to follow up points raised in the text without making it unduly cumbersome as an introductory text.

S. Schor,
Dept. Medical Oncology,
Christie Hospital & Holt Radium Institute,
Manchester